# Characterizing household air pollutant concentrations associated with an electrification program in the rural San Joaquin Valley

**DOI:** 10.1088/2752-5309/ae4ac9

**Published:** 2026-03-25

**Authors:** Michael Johnson, Katherine A Kearns, Jesus Rivera, Sydney M Jones, Jessica Tryner, Heather Miller, Ahana Ghosh, Maria Fe Aragon, Misbath Daouda, Ajay Pillarisetti, Tim Tyner

**Affiliations:** 1 Berkeley Air Monitoring Group, Berkeley, CA, United States of America; 2 Central California Asthma Collaborative, Fresno, CA, United States of America; 3 Access Sensor Technologies, Fort Collins, United States of America; 4 University of California, Berkeley, United States of America

**Keywords:** indoor air quality, nitrogen dioxide, gas stoves, induction stoves, low-cost sensors, asthma

## Abstract

Indoor nitrogen dioxide (NO_2_) and fine particulate matter (PM_2.5_) are concerns in U.S. households, especially those that cook using gas or propane stoves. Exposures to these and other indoor pollutants are linked to a variety of adverse health outcomes, including asthma morbidity, that disproportionately affect low-income households. We conducted a cross-sectional study of 138 homes in four low-income rural communities in California’s San Joaquin Valley, comparing air pollutant concentrations between households that participated in a state electrification program and households using propane or natural gas for cooking. In each home, pollutants were monitored for approximately one month using personal air monitors and for 48 h using reference-grade instruments. Median 48-h average indoor NO_2_ concentrations were 63% lower in electric stove homes (electric: 6.0 ppb, gas: 16.0 ppb, *p* < 0.001). No electric stove homes had 48-h indoor NO_2_ concentrations exceeding the California annual guideline of 30 ppb, while 17% of gas homes did. Additionally, no electric stove homes had 1-h rolling-average NO_2_ concentrations exceeding the 100-ppb level deemed unhealthy for sensitive groups by the U.S. Environmental Protection Agency, whereas 41% of gas homes exceeded this threshold. PM_2.5_ concentrations were similar across groups, indicating that cooking-related emissions from food were the dominant contributor to PM_2.5_ mass concentrations rather than particles generated from gas combustion. Our evaluation of monitoring durations showed that two to four days of NO_2_ data and one week of PM_2.5_ data provided reliable estimates of longer-term averages, suggesting that shorter campaigns may yield robust estimates of indoor air quality. These results support the provision of electric cooking technologies as a strategy to address air quality-related health risks in rural, low-income communities and provide new evidence from an understudied population that can inform future indoor air quality research and energy transition policies.

## 1. Introduction

Concerns about the health risks of gas stoves have grown in recent years, and related policy discourse has become more prevalent [1–4]. Approximately 38% of U.S. households, and more than 70% of households in California, cook with gas ranges, cooktops, or ovens [5]. These appliances emit nitrogen dioxide (NO_2_), carbon monoxide (CO), and other combustion byproducts, including carcinogenic compounds such as benzene, directly into the indoor environment, where concentrations of these pollutants can quickly exceed health-based guidelines [6–10]. For example, controlled chamber and field measurements in California found that gas stoves can rapidly push indoor NO_2_ above the 1-h United States Environmental Protection Agency (EPA) standard of 100 ppb, especially in smaller kitchens without effective ventilation [8, 9]. Prior work links exposure to air pollutants emitted by gas cooking burners with asthma, cardiovascular disease, and other adverse health outcomes, particularly in children and other vulnerable populations [7, 11–14]. Unlike furnaces or water heaters, gas stoves are often installed without external ventilation. Even kitchen venting hoods, which show benefits under controlled conditions [8, 15], do not always provide clear indoor air quality benefits in real-world settings [16].

Policy responses to these risks are accelerating. More than 130 jurisdictions across the United States have adopted restrictions or bans on new gas connections, motivated by public health and climate-related concerns [17, 18]. At the same time, these measures have faced legal and political challenges. In August 2025, for example, the Association of Home Appliance Manufacturers filed suit in Colorado to block a requirement for informational labels on gas stoves that would guide consumers to resources on how they affect indoor air quality [19, 20]. Within this context, electrification programs provide an opportunity to generate empirical evidence on the potential indoor air quality benefits of replacing gas with electric stoves, which in turn can help guide policymaking and inform consumer choices and behaviors.

In California, the San Joaquin Valley, which is characterized by high poverty rates and a legacy of fossil fuel dependence, ranks at or above the 90th percentile for pediatric asthma emergency room visits statewide [21]. Valley residents experience significant exposure to PM_2.5_ and NO_2_ from residential fuel use, agricultural activities, wildfire smoke, and traffic, all of which contribute to elevated rates of respiratory diseases, including asthma and chronic obstructive pulmonary disease [22, 23]. Air quality-related health impacts can be especially pronounced in rural communities, where infrastructure limitations and reliance on polluting fuels contribute to persistent indoor air quality challenges.

Previous studies have demonstrated that replacing gas stoves with electric alternatives improves indoor air quality. In Boston public housing, residents who moved into ‘green’ units with electric stoves experienced 57% lower indoor PM_2.5_ and 65% lower NO_2_ concentrations, alongside improved self-reported health outcomes, compared with residents of conventional units [24]. More recently, a pilot in New York City public housing documented a 56% reduction in weeklong average indoor NO_2_ after gas stoves were replaced with induction stoves. In that study, controlled cooking tests showed that gas burners drove kitchen NO_2_ from background levels (near 18 ppb) to peaks exceeding 190 ppb, while induction cooking did not increase indoor NO_2_ pollution above background levels [10]. These prior programs and studies focused exclusively on urban settings; however, it is important that rural U.S. households are not excluded from the potential indoor air quality benefits of electrification.

This study, based in the rural San Joaquin Valley, compares indoor levels of NO_2_, NO, NO*_x_*, CO, CO_2,_ fine particulate matter (PM_2.5_), and black carbon (BC) between homes in a California Public Utilities Commission (CPUC) electrification program that provided electric stoves and control homes with gas stoves. Here we present the study’s main results, focusing on how these pollutant concentrations differed between CPUC program homes with electric stoves and control homes with continued gas stove usage.

## 2. Methods

### 2.1. Study design overview

This study used a cross-sectional design to compare indoor air quality in homes that participated in a CPUC electrification program with similar homes using propane or natural gas for cooking (henceforth referred to as gas). In total, 139 households were recruited, including 66 CPUC program homes using electric stoves and 73 homes using gas for cooking. Indoor concentrations of NO_2_, CO, CO_2_, and PM_2.5_ were monitored over approximately one month using low-cost air monitors (henceforth referred to as the ‘longer-term’ monitoring period), complemented with 48-h deployments of reference NO_2_ and PM_2.5_ instruments to validate and calibrate the low-cost sensor data. Information on household characteristics, ventilation, and appliance use was collected through observation and with surveys to control for potential confounders. Each home was also provided with a smart, low-cost air filtration device approximately halfway through monitoring to assess its potential to reduce indoor PM_2.5_ levels, particularly under wildfire smoke conditions. The smart filtration device was a box fan (2000 CFM) with a 3M Filtrete MERV13 filter fixed to the intake, and equipped with a Plantower PMS5003 sensor that activated the fan when PM_2.5_ exceeded 12 *μ*g m^−3^ (U.S. EPA annual standard at time of study). Participants used the fan as desired, and were not obligated to keep it plugged in or installed in a specific room. Further details on the smart filtration device, including methods, analysis, and results, are provided in Supplementary Material (SM) Section 6, as interpretation of these results is limited by uncertainty in device usage and the relatively mild wildfire conditions during the monitoring period.

### 2.2. Site and home selection criteria

The study was conducted from August 2022 through January 2024 in four rural communities in the San Joaquin Valley: Alpaugh, Allensworth, Ducor, and West Goshen. The area is characterized by high rates of propane use for cooking and heating, as well as high rates of respiratory illness [21], environmental exposure risks [25], and wildfire-related air pollution [22], making it a critical focus area for air quality intervention efforts. The CPUC’s San Joaquin Valley Disadvantaged Communities pilot program launched in 2018 and targeted rural communities without natural gas service [26]. Communities eligible for this pilot program had at least 100 residents and had at least 25% of households enrolled in CARE (California Alternate Rates for Energy, which provides a 30%–35% discount on energy bills for households at or below 200% of the Federal Poverty Guidelines) or FERA (Family Electric Rate Assistance, which provides an 18% discount for larger households slightly above the CARE threshold) [27]. CPUC program homes received no-cost electrification upgrades such as heat pumps, induction stoves, and electric dryers to improve affordability, health, and air quality. Homes were eligible for participation in the air quality study if they met the following criteria: (1) used gas for cooking (control group) or used an electric stove and were in the CPUC program, (2) were located within the study communities, and (3) were willing to allow installation of monitoring equipment. Recruitment was carried out with support from the Center for Race, Poverty, and the Environment (CRPE), a local community organization partnering in the CPUC program. CRPE helped our field staff build connections in the community and reach potential participants through community events, phone calls, and door-to-door outreach. The study protocol was reviewed and approved by the Advarra Institutional Review Board (Protocol #Pro00065195).

### 2.3. Pollutant and environmental measurements

Longer-term indoor air quality was monitored in each home using the Personal Air Monitor (PAM, 2B-Tech, USA) [28], which contains a light-scattering sensor for PM_2.5_ (Plantower PMS7003), electrochemical sensors for NO_2_ (Alphasense A43F) and CO (Alphasense CO-A4), and a non-dispersive infrared sensor for CO_2_ (Telaire T6713). The PAMs were placed in the kitchen at approximately 1–1.5 m horizontally from the stove, 1.5–2 m above the floor, and, to the extent possible, away from doors and windows. During the longer-term monitoring period, an intensive 48 h of sampling (generally in the middle two weeks) was conducted by co-locating the PAM with: (1) an Ultrasonic Personal Air Sampler (UPAS v2.1 PLUS, Access Sensor Technologies, USA) [29] to collect filter samples of PM_2.5_ and (2) a Model 405 Monitor (2BTech) [30] for time-resolved NO_2_, NO, and NO*_x_* measurements. The UPAS and 405 Monitor provided reference-quality PM_2.5_ and NO_2_ measurements and were used for independent analyses and to correct the co-located PAM’s PM_2.5_ and NO_2_ sensor data. The 405 Monitor measures NO_2_ by direct absorption of 405 nm light and is approved by the U.S. EPA as a federal equivalent method for regulatory NO_2_ monitoring [30]. This instrument was factory-calibrated at the onset of the study and zeroed before each deployment. See the SM section 1 for more details on how the PAM NO_2_ sensor data were corrected using Model 405 NO_2_ data, how the PAM CO and CO_2_ sensors were calibrated with gas standards, and how the PAM CO_2_ data were used to estimate ventilation rates (SM section 3).

The longer-term monitoring also included measurements of stove and venting hood usage. In each control home, an Aranet4 CO_2_ and temperature logger was placed directly above the gas stove to monitor use by capturing peaks in CO_2_ concentrations associated with combustion events (defined as 2000 ppm or higher). Aranet4 devices were also placed above the electric stoves to identify use events via temperature peaks. Each venting hood was checked observationally and categorized as recirculating (venting back into the kitchen) or extractive (venting outdoors). In homes with extractive hoods that vented outdoors, a small anemometer was affixed to the intake, with any air velocity greater than 0.25 m s^−1^ logged as the hood being used. We did not attempt to estimate extraction flow rates from anemometer measurements because of between-home variability in hood design, ducting, and measurement geometry, and participant recall of typical fan speed was not collected due to concerns about reliability and recall bias.

These physical measurements were complemented with surveys given to participants by trained field staff to obtain information on home characteristics, including home size (square feet), building materials, ventilation systems, and potential indoor pollution sources (e.g., smoking, candles, or incense).

Additionally, ambient PM_2.5_ concentrations were assessed using UPAS installed at school sites in Allensworth, Alpaugh, and Ducor. Each UPAS was installed at approximately three to four meters above the ground in an environmental enclosure for protection from rain and direct sunlight. Two-week ambient PM_2.5_ samples were collected continuously at each school throughout the study. When the study was expanded to West Goshen to collect additional household samples, resources were not available for additional ambient measurements. We supplemented ambient data from the regulatory PM_2.5_ monitor nearest to West Goshen, which was downloaded from the U.S. EPA air quality system (site ID=06-107-2003 in Visalia, and see additional information in SM section 7).

All filters were stored at −20 °C until shipment to Colorado State University (Fort Collins, CO, USA), where PM_2.5_ and BC analyses were performed using an automated robotic system [31]. PM_2.5_ mass was determined gravimetrically with a microbalance (XS3DU, Mettler Toledo, USA; 0.1-*µ*g resolution) under controlled temperature (20 °C) and humidity (40% RH) conditions, following U.S. EPA reference protocols. Each filter was weighed three times before and after sampling. BC was quantified by measuring the change in optical transmittance of filters at 880 nm with a SootScan OT-21 Transmissometer (Magee Scientific, USA) and converted to mass following the approach adopted in the Household Air Pollution Intervention (HAPIN) Trial (mass absorption cross section of 13.7 m^2^ g^−1^) [32–34]. We had 21 blank filter samples with median PM and BC mass depositions of 4.7 *μ*g and 3.6 *μ*g, respectively. Limits of detection (LoD) were estimated as three times the standard deviation of blank filter values for PM_2.5_ (16.9 *μ*g) and BC (10.7 *μ*g). PM_2.5_ depositions below the LoD (*n* = 15) were substituted with the LoD divided by 2^0.5^. Adjusting for the relatively high BC field blank deposition estimates, especially given that the PM_2.5_ depositions for the respective filters were low, would have resulted in 113 filters having their BC values replaced. We present results here with and without blank adjustment for the BC filters (see SM section 2 for additional methods and results for BC). Concentrations (*µ*g m^−3^) were calculated by dividing the mass depositions by the sample volume. For analysis, only samples with valid metadata and a duration within four hours of a 24-h period were included (e.g., 20–28 h, 44–52 h, 68–76 h, 92–100 h); filters failing these criteria were excluded (*n* = 24).

### 2.4. Data analysis

Descriptive summary statistics were calculated for indoor concentrations of primary pollutants measured in each home during the longer-term monitoring period, the 48-h intensive monitoring period, and the smart filter evaluation period by study arm. Calibrated and corrected concentrations of NO_2_, CO, CO_2_, and PM_2.5_ from the PAM were generated for the longer-term measurement duration and the 48-h period during which the UPAS and Model 405 were co-located with the PAM. Concentrations of PM_2.5_, NO, NO_2_, and NO*_x_* measured using the UPAS and Model 405 were assessed for the 48-h intensive monitoring period. One-hour moving averages of the 405 NO_2_ data were also calculated for comparison against U.S. EPA 1-h air quality guidelines and standards, including the air quality index (Good: 0–53 ppb, Moderate: 54–100 ppb, Unhealthy for Sensitive Groups: 101–360 ppb, Unhealthy: 361–649 ppb, Very Unhealthy: 650–1249, and Hazardous: ⩾1249 ppb) [35] and the 1-h National Ambient Air Quality Standard (100 ppb) [36]. Given the non-normal distribution of the data, Wilcoxon rank sum tests were used to determine if there were statistical differences between pollutant concentrations by study arm.

An analysis of monitoring duration was conducted by comparing cumulative daily averages of NO_2_ and PM_2.5_ against a 21 d reference mean in homes with at least three weeks of data. For each household, cumulative means were calculated for successive days and assessed using Pearson correlation coefficients and mean bias relative to the 21 d average.

Home ventilation rates (quantified as air change rates per hour [ACH]) were estimated by applying a mass balance to each home and evaluating the rate of change in the indoor CO_2_ concentration during decay events [37]. Additional information is provided in SM section 3. In most homes, multiple estimates of the ventilation rate were obtained from multiple CO_2_ decay events that were identified in the longer-term PAM’s data. These estimates were used to calculate the median ventilation rate for each home.

Machine learning-based least absolute shrinkage and selection operator (LASSO) regression models were used to assess the association between the electric stove group (CPUC program homes) and NO_2_ (405 monitor) and PM_2.5_ (UPAS) concentrations during the intensive monitoring period, accounting for the clustering of covariates within households and communities. LASSO methods perform variable selection and coefficient estimation simultaneously using a shrinkage approach. The technique reduces the coefficients of less important predictors to zero based on the sum of squared residuals, which addresses overfitting in high-dimensional models [38–40]. Models were trained using 70% of the dataset, with 10-fold cross-validation used to determine the optimal penalty term (*λ*), based on the minimum cross-validated mean squared error (MSE). Variables retained by LASSO were refitted in an ordinary least squares (OLS) model for interpretability, known as post-LASSO [41–43]. Primary outcomes of 48-h PM_2.5_ (UPAS) and NO_2_ (405) were log-transformed. Covariates, including seasonality, smart filter presence, ventilation rates, home characteristics (e.g., number of people living in the home, square footage, kitchen style, smokers present, pets present), appliance usage, and participant behaviors during the final 24 h of the 48-h intensive sampling period (e.g., frequency that windows and doors were open, non-cooking sources of air pollution) were applied to control for potential confounders. Multicollinearity among predictors was assessed using pairwise correlation coefficients, with variables exceeding a correlation coefficient of 0.6 examined and selected based on assumed relevance. Continuous predictor variables were standardized, and households with missing data were removed. As sensitivity analyses, we fitted both a full multivariate linear regression model including all covariates and a stepwise regression model for comparison. Model performance was compared based on MSE, root MSE, mean absolute error, *R*^2^, and normalized MSE. Statistical analysis and model development were performed using R software (version 4.4.2).

## 3. Results

### 3.1. Sample summary and household characteristics

A total of 139 households were initially enrolled; one home dropped out of the study, resulting in a total of 138 homes included in the analysis (table 1). There were 72 homes (52%) in the electric stove group and 66 homes (48%) in the gas stove group. The distribution of study groups was generally balanced across all four communities. The control group included a slightly higher proportion of households in Allensworth (61%), Alpaugh (56%), and Ducor (51%) compared to the electric stove group, with West Goshen being the exception (31%). The electric stove group used primarily induction stoves installed through the CPUC program (94%). A small number of homes (*n* = 4) received other electric appliances through the program (e.g., heat pumps), but already had smooth-top resistance-style electric stoves. The control group used propane (82%) or natural gas (18%) for cooking. Only two homes reported using pellet stoves for heating, and fireplaces (*n* = 6), even when present, were reportedly used rarely (only two households reported using a fireplace). Heating and cooling technologies differed by study arm, with the electric stove group using more heat pumps (68%) than the control group (7.5%) due to their provision from the CPUC program. The study arms were well-matched in household characteristics, including household size, education levels, income distribution, square footage, venting hoods, and smoking prevalence.

**Table 1.** Household characteristics summarized by study arm and overall.

	Gas stove group	Electric stove group	Overall
	*N* = 66	*N* = 72	*N* = 138
Community			
Allensworth	19 (29%)	12 (17%)	31 (22%)
Alpaugh	14 (21%)	11 (15%)	25 (18%)
Ducor	19 (29%)	18 (25%)	37 (27%)
West Goshen	14 (21%)	31 (43%)	45 (33%)
Average number of people in the household	4.6 (2.3)	3.9 (1.8)	4.2 (2.1)
Approximate square footage of household			
1000 or less square feet	29 (44%)	32 (44%)	61 (44%)
1000–2000 square feet	37 (56%)	39 (54%)	76 (55%)
2000–3000 square feet	0 (0%)	1 (1.4%)	1 (0.7%)
3000 or more square feet	0 (0%)	0 (0%)	0 (0%)
Highest education level reached by anyone in household			
Either formal education or secondary education is not complete	16 (24%)	15 (21%)	31 (22%)
Secondary education completed	29 (44%)	31 (43%)	61 (43%)
Associate’s or vocational degree completed	10 (15%)	14 (19%)	24 (17%)
Bachelor’s degree completed	10 (15%)	10 (14%)	20 (14%)
Graduate school degree completed	1 (1.5%)	2 (2.8%)	3 (2.2%)
Total household income per year			
$20 000 or less	23 (35%)	18 (25%)	41 (30%)
$20 000 to $50 000	23 (35%)	31 (43%)	54 (39%)
$50 000 to $100 000	4 (6.1%)	12 (17%)	16 (12%)
$100 000 or more	1 (1.5%)	1 (1.4%)	2 (1.4%)
Decline to answer	15 (23%)	10 (14%)	25 (18%)
Does anyone in the household smoke?			
No	58 (88%)	62 (86%)	120 (87%)
Yes	8 (12%)	10 (14%)	18 (13%)
Kitchen style			
Galley	5 (7.6%)	5 (6.9%)	10 (7.2%)
Open	52 (79%)	58 (81%)	110 (80%)
Own room	9 (14%)	9 (13%)	18 (13%)
Stove technology			
Induction electric	0 (0%)	68 (94%)	68 (49%)
Natural gas	12 (18%)	0 (0%)	12 (8.7%)
Propane	54 (82%)	0 (0%)	54 (39%)
Resistance electric (smooth-top)	0 (0%)	4 (5.6%)	4 (2.9%)
Ventilation hood above stove			
No	20 (30%)	17 (24%)	37 (27%)
Yes	46 (70%)	55 (76%)	101 (73%)
Venting hood exhausts to the outside			
No	3 (6.5%)	9 (16%)	12 (12%)
Yes	43 (93%)	46 (84%)	89 (88%)
Current Heating Technology^a^			
Propane	14 (21%)	0 (0.0%)	14 (10%)
Wood/pellet Stove	2 (3.0%)	0 (0.0%)	2 (1.4%)
Wood fireplace	5 (7.5%)	1 (1.4%)	6 (4.3%)
Natural gas	7 (10%)	0 (0.0%)	7 (5.0%)
Resistance electric	26 (39%)	20 (27%)	46 (33%)
Heat pump	5 (7.5%)	50 (68%)	55 (39%)
Other	4 (6.0%)	2 (2.7%)	6 (4.3%)
None	4 (6.0%)	0 (0.0%)	4 (2.9%)
Current Cooling Technology^a^			
Window A/C	18 (25%)	0 (0.0%)	18 (12%)
Conventional central A/C	29 (41%)	36 (48%)	65 (45%)
Heatpump	3 (4.2%)	35 (47%)	38 (26%)
Swamp cooler	16 (23%)	1 (1.3%)	17 (12%)
Portable A/C	0 (0.0%)	1 (1.3%)	1 (0.7%)
Fan	4 (5.6%)	2 (2.7%)	6 (4.1%)
None	1 (1.4%)	0 (0.0%)	1 (0.7%)
			
No	34 (52%)	32 (44%)	66 (48%)
Yes	32 (48%)	40 (56%)	72 (52%)
Bathroom ventilation fan can be turned off			
No	1 (3.1%)	3 (7.5%)	4 (5.6%)
Yes	31 (97%)	35 (88%)	66 (92%)
Other	0 (0%)	2 (5.0%)	2 (2.8%)
Air filter/purifier present at house (prior to provision of smart filter)			
No	65 (98%)	67 (93%)	132 (96%)
Yes	1 (1.5%)	5 (6.9%)	6 (4.3%)

^a^ Multiple choices could be selected.

### 3.2. Pollutant concentrations

**Figure 1.**
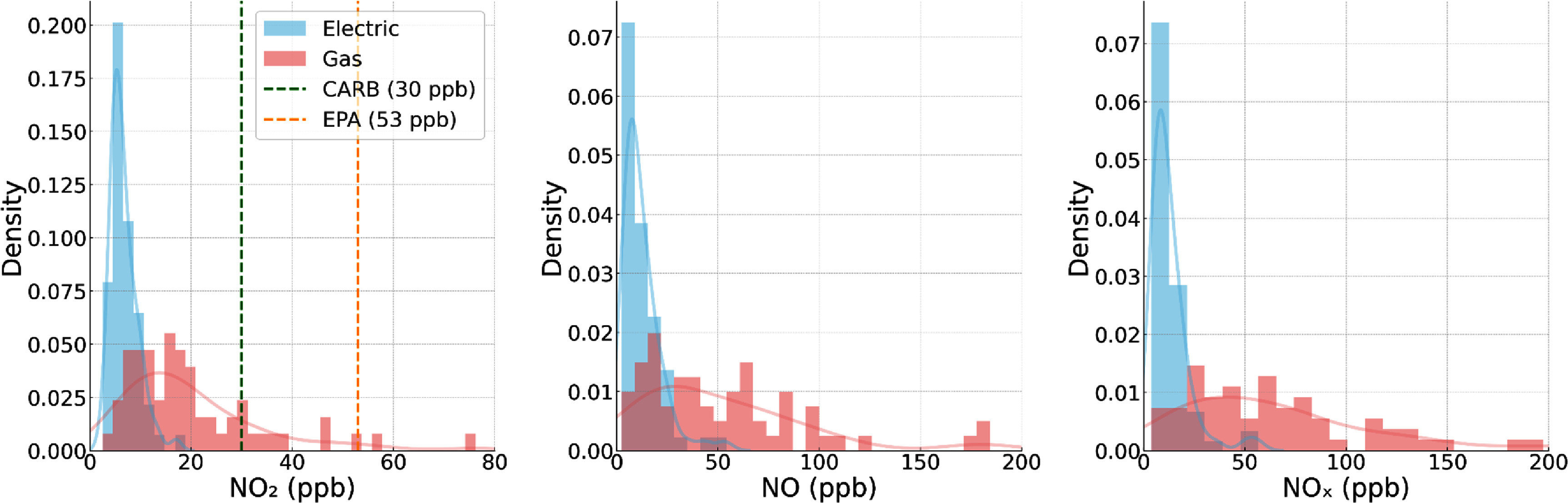
Distributions of the 48-h time-averaged indoor nitrogen oxide concentrations during the intensive monitoring period by study group. Histograms with density overlays show measured NO_2_ (left), NO (center), and NO*_x_* (right) in homes with electric (blue) and gas (red) cooking. Vertical dashed lines on the NO_2_ panel mark the CARB annual guideline (30 ppb, green) and U.S. EPA annual standard (53 ppb, orange).

Summary statistics from intensive 48-h monitoring with reference instruments and PAMs, and longer-term PAMs monitoring are presented in table 2 and figure 1 (additional box plots are presented in SM figures S2-S4). Longer-term monitoring averaged 26 (*σ* = 9) days of available data. Median indoor concentrations of combustion-related nitrogen oxides were lower in homes with electric stoves compared to those with gas stoves. During intensive monitoring periods, the 405 Monitor measured median 48-h average NO_2_, NO, and NO*_x_* concentrations of 6.0, 9.3, and 9.5 ppb in electric stove homes versus 16.0, 39.7, and 59.1 ppb in gas stove homes, representing reductions of 63%, 76%, and 84%, respectively (all *p* < 0.001). No electric stove homes had a 48-h average NO_2_ concentration above the California Air Resources Board annual guideline of 30 ppb [44] during intensive monitoring, whereas 17% of gas stove homes had 48-h average NO_2_ concentrations exceeding this threshold (figure 1). For the longer-term PAM measurements, median NO_2_ was 4.5 ppb in electric homes compared to 16.7 ppb in gas homes, corresponding to a 73% reduction (*p* < 0.001). These time-averaged NO_2_ concentrations are compared to annual guidelines (rather than 24-h guidelines) because these 48-h and longer-term averages are assumed to represent the NO_2_ concentrations residents are chronically exposed to throughout the year; similar assumptions are common for indoor air quality and exposure studies [45–49].

**Table 2.** Summary statistics of time-averaged NO_2_, NO, NO*_x_*, CO, CO_2,_ PM_2.5_, and BC concentrations as well as air change rates per hour (ACH). Significant differences are indicated by bold text and determined using two-sided Wilcoxon rank-sum tests. Differences are reported as percent changes in medians; this interpretation assumes the groups have similarly shaped distributions.

	Reference Monitors (∼48 h)					PAMs (∼48 h)				PAMs (longer-term)				ACH (longer-term)
	NO_2_ ppb	NO ppb	NO*_x_* ppb	PM_2.5_ *µ*g m^−3^	BC *µ*g m^−3^	NO_2_ ppb	CO ppm	CO_2_ ppm	PM_2.5_ *µ*g m^−3^	NO_2_ ppb	CO ppm	CO_2_ppm	PM_2.5_ *µ*g m^−3^	h^-1^
GAS														

*n*	52	52	52	51	51	49	37	55	52	49	40	56	53	53
Mean	29.8	54.0	66.6	24.2	1.3	20.1	1.3	1173	13.4	21.8	1.4	1170	16.6	0.56
SD	13.9	42.9	48.7	23.8	0.9	16.5	1.4	771	14.0	16.3	1.3	754	18.6	0.73
Median	16.0	39.7	59.1	14.8	1.2	15.4	0.8	1014	9.2	16.7	1.0	1019	10.3	0.34
Maximum	78.4	238.2	248.6	126.1	5.4	72.3	4.4	5313	130.9	85.5	6.2	5395	116.3	

ELECTRIC														

*n*	66	66	66	62	62	44	40	50	51	44	49	61	61	59
Mean	6.7	12.6	12.9	15.0	1.1	8.5	0.7	973	12.3	8	0.8	960	12.7	0.29
SD	2.7	9.6	9.9	11.2	0.9	8.9	0.8	428	9.8	7.6	0.7	402	6.9	0.15
Median	6.0	9.3	9.5	13.0	0.9	5.7	0.4	856	8.6	4.5	0.5	893	11.0	0.26
Maximum	25.5	74.9	90.8	149.9	5.2	41.2	3.9	2415	35.6	31.8	2.7	2397	34.1	
Difference in electric home median	−63%	−76%	−84%	−12%	−25%	−63%	−50%	−16%	−8%	−73%	−50%	−12%	7%	−27%
*p*-values	**<0.001**	**<0.001**	**<0.001**	0.06	0.16	**<0.001**	**0.02**	0.11	0.85	**<0.001**	**0.02**	0.06	0.9	**0.008**

Average 48-h NO_2_ concentrations measured using PAMs during intensive monitoring were strongly correlated with longer-term time averages (Pearson *r* = 0.89) with negligible bias (<1 ppb), indicating the shorter-term measures were representative of longer-term concentrations (see figures S6 and S7 in the SM). Additional analyses of assumed increasing sampling durations (see SM section 4) showed that, for NO_2_, correlations between the cumulative time average and the 21-d time average (minimum threshold of data availability for sampling duration analysis) exceeded a Pearson r of 0.9 after four days in electric homes and just two days in gas homes, with bias remaining low (<10% or <1 ppb). For PM_2.5_, correlations rose more slowly, reaching *r* > 0.9 after 7–10 d and stabilizing above 0.95 within two weeks. Bias was generally within ±5% within the first 10 days and narrowing to ±1%–2% after 10 d.

Median time-averaged PM_2.5_ concentrations were similar across stove types. For the intensive reference monitoring, median 48-h averages were 13.0 *µ*g m^−3^ in electric homes and 14.8 *µ*g m^−3^ in gas homes (−12% in electric homes, *p* = 0.06), while median longer-term averages calculated from PAMs data were 11.0 and 10.3 *µ*g m^−3^, respectively (+7% in electric homes, *p* = 0.90). Median 48-h average BC concentrations were also similar between electric and gas homes (0.9 vs 1.2 *µ*g m^−3^; −25% in electric homes, *p* = 0.12), which was expected, as prior studies have indicated that most indoor PM_2.5_ mass arises from cooking of food rather than gas combustion [50–53].

Median time-averaged CO over the longer-term monitoring period was lower in electric homes (0.5 vs 1.0 ppm, −50%). Median time-averaged concentrations in both groups were well below the California 24-h guideline of 3.5 ppm, though time-averaged CO concentrations greater than 4 ppm were measured in two gas homes, which suggested specific instances of poorer gas combustion or other CO sources. For CO_2_, median time-averaged concentrations over the longer-term monitoring period were 893 ppm in electric homes versus 1019 ppm in gas homes (−12%, *p* = 0.06), consistent with combustion as a source of CO_2_.

Median ventilation rates in homes with electric and gas stoves were 0.26 h^–1^ and 0.34 h^–1^, respectively (see SM section 3 for more detailed results). A total of 91 homes had extractive hoods; however, only 34 electric homes and 29 gas homes had available usage rate data due to anemometer supplier delays. Median usage rates were 32.1 min d^−1^ for electric stove homes and 29.4 min d^−1^ for gas stove homes (*p* = 0.92 using the Wilcoxon rank-sum test).

### 3.3. Impact of covariates on NO_2_

**Figure 2.**
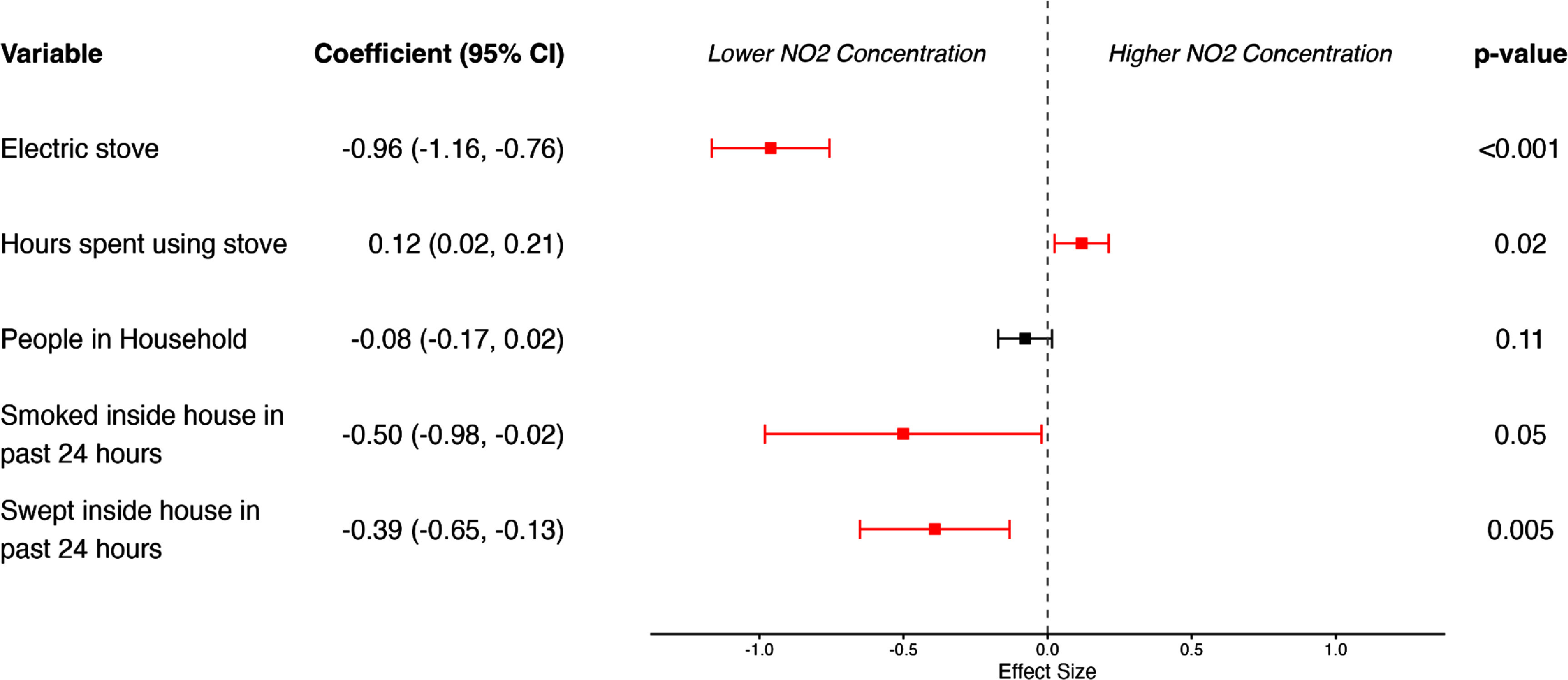
Forest plot of ordinary least squares regression estimates from LASSO-selected variables associated with indoor NO_2_ concentration. Coefficient (*β*) estimates and 95% confidence intervals are displayed to the left for each predictor, and *p*-values are displayed to the right of each predictor. The dashed vertical line represents the null effect (*β* = 0). *Note:* The OLS model was fitted only to the training subset of the data (70% of total observations) using the variables selected by the LASSO procedure.

Of the 25 potential covariates considered for inclusion in the OLS model for NO_2_, five were retained by the LASSO procedure (see figure 2). Being in the electric stove group, self-reported smoking in the house during the final 24 h of the intensive monitoring period, and self-reported sweeping the house during the same 24-h period, were all significantly associated with lower NO_2_ concentrations. The effect size was largest for being in the electric stove group. Conversely, self-reported hours spent cooking with the stove in the past 24 h were associated with higher NO_2_ concentrations. Having more people in the home appeared to be associated with lower NO_2_ concentrations as well, but this association was not significant. A model for PM_2.5_ was also developed, and it retained only kitchen ventilation parameters (windows and doors being opened) by LASSO (see SM figure S11). The sensitivity analyses of the full and stepwise models, including models that accounted for the anemometer-based direct measures of venting hood use, are presented in supplementary material (see SM section 5 and tables S2–S7).

### 3.4. Peak NO_2_ concentrations and cooking patterns

**Figure 3.**
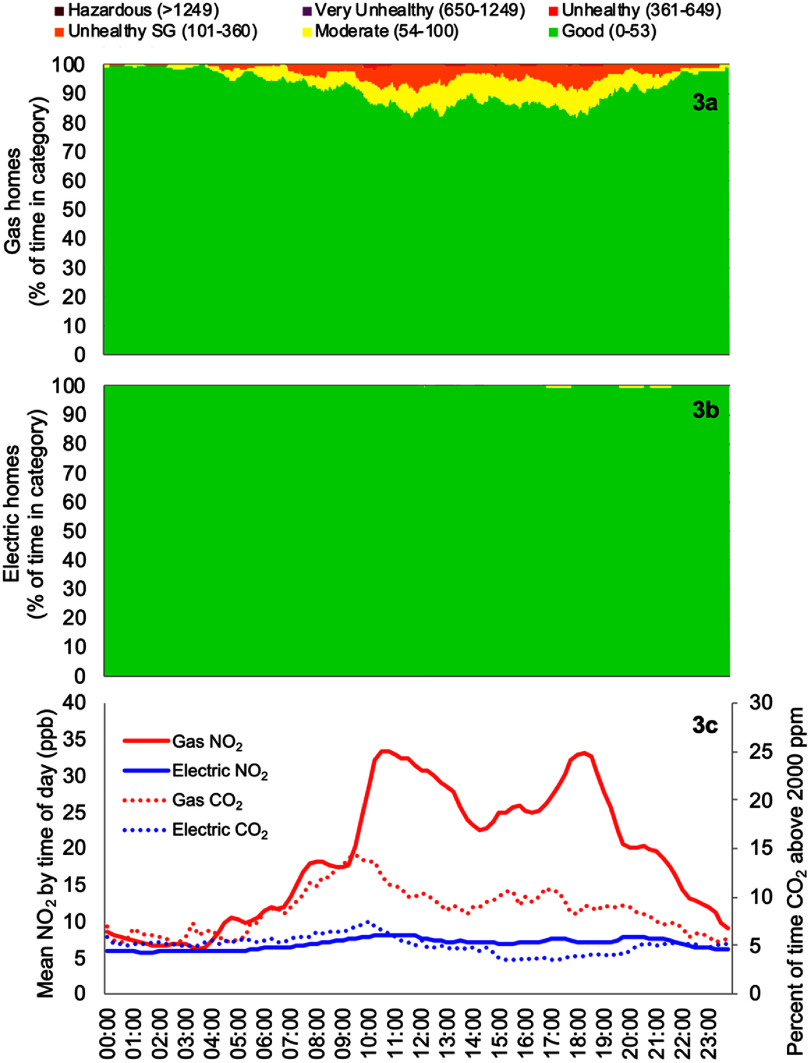
Time-resolved indoor air pollutant concentrations by study group (gas and electric stoves). (3a) and (3b): Distributions showing the percentage of time at each minute of the day with NO_2_ concentrations at each AQI category. (3c): Mean NO_2_ concentrations (solid lines) by time of day for each study group (primary *y*-axis), with percentage of time where CO_2_ measured above the stove exceeded 2000 ppm (dotted lines and secondary *y*-axis).

To further characterize indoor pollutant patterns, similar to previous analyses of time-resolved indoor air pollution [54–56], NO_2_ measurements from the 405 monitor were analyzed as a time series depicting the corresponding U.S. EPA air quality index (AQI) category for each minute of the day (see figure 3) [35]. Consistent with U.S. EPA procedures, each minute’s AQI classification was determined from the 1-h moving average of NO_2_ concentrations. Data from all monitoring days were then aggregated to produce an average daily profile by study group. In homes with gas stoves, exceedances of the U.S. EPA 1-h standard (100 ppb), corresponding to the ‘Unhealthy for Sensitive Groups’ category, were observed in up to ∼10% of homes at peak times. In contrast, electric stove homes remained almost entirely within the ‘Good’ AQI range, with only minor excursions into ‘Moderate,’ and had no intervals exceeding 100 ppb. Overall, gas stove homes exceeded the ‘Good’ category threshold (53 ppb) for 8% of each day, on average, compared to less than 0.1% for electric stove homes. The highest 1-h moving average recorded in gas stove homes was 528 ppb, compared to a maximum of 72 ppb in electric stove homes.

In the lower panel, the NO_2_ time series presents the aggregated mean concentrations across all homes within each study arm, highlighting the contrast between gas and electric stove households. The mean trace from gas stove homes had distinct diurnal peaks of approximately 30–35 ppb during morning and evening cooking periods, whereas the electric stove home trace remained below 10 ppb. In gas stove homes, CO_2_ concentrations measured directly above the cooking surface, which served as a proxy for combustion-based stove use, exceeded 2000 ppm more frequently during morning and evening cooking periods, coinciding with higher NO_2_ concentrations. This correspondence indicated that gas combustion was the source of concurrent increases in both CO_2_ and NO_2_. In contrast, electric stove homes showed neither elevated CO_2_ nor NO_2_ concentrations, consistent with the absence of combustion emissions. Cooking event temperature peaks from electric stove use were deemed too low or indistinct to be identified reliably and thus were not analyzed for the electric stove homes.

## 4. Discussion

### 4.1. Comparison to findings from prior studies

Our findings add to a growing body of evidence that gas cooking is associated with higher indoor NO_2_ concentrations, while homes using electric stoves have lower concentrations (SM section 8). In this study, households with propane or gas stoves had a median 48-h average NO_2_ concentration of 16 ppb compared with 6 ppb in electric homes (63% difference). The corresponding difference in median longer-term NO_2_ concentrations was 16.7 ppb versus 4.5 ppb (73% difference). Whereas 17% of gas homes had 48-h average indoor NO_2_ concentrations that exceeded the CARB annual guideline of 30 ppb, no electric homes did. Gas homes also had peak indoor NO_2_ concentrations exceeding the U.S. EPA 1-h standard of 100 ppb, whereas such exceedances were not observed in electric homes. In-home and chamber studies in California have shown that gas cooking burners can raise indoor NO_2_ above 100 ppb within minutes [8, 9, 57].

These findings are consistent with a broad range of prior studies that have reported ∼35%–70% lower indoor NO_2_ concentrations in homes with electric or induction stoves compared with gas stoves. Indoor NO_2_ concentrations measured in studies with electric and gas stove comparisons are illustrated in table S10. For example, Colton *et al* [24] found 65% lower concentrations in Boston ‘green’ housing with electric appliances, and Daouda *et al* [10] observed a 56% decrease in New York City public housing following gas-to-induction stove replacements. Multi-state and international analyses reinforce this pattern, with measured decreases in daily indoor NO_2_ levels ranging from 50%–65% across the U.S [7], 65%–70% in Canada [58], and 40%–60% in European case studies [59]. The 63%–73% lower time-averaged indoor NO_2_ levels observed in our electrified study homes fall within the upper end of this previously reported range, adding weight to evidence that electrification is consistently associated with lower indoor NO_2_ concentrations across contexts. Our findings extend the knowledge base by demonstrating this pattern in a relatively large sample (138 homes) with long-term monitoring (four weeks per home) in rural, low-income communities in California’s San Joaquin Valley, a population type largely absent from prior research despite its elevated asthma prevalence and disproportionate respiratory health burden [22, 25].

### 4.2. Improving household air quality

The study arm comparisons and models showed that homes with electric stoves had lower indoor NO_2_ concentrations than those with gas stoves, reinforcing evidence in the literature that transitioning from gas to electric reduces indoor concentrations of combustion-derived pollutants [8, 10, 24, 57, 58]. Interestingly, we found no significant associations between air exchange rate and indoor concentrations of NO_2_ or PM_2.5_. We also ran the model separately with the direct vent hood use rates, given the small sample size, but observed no significant effect of directly measured extractive kitchen vent hood use time, although the effect was in the expected direction (lower concentrations with increased use rates). Conversely, the model indicated lower NO_2_ in homes that reported sweeping and smoking, activities which should have no or minimal direct impact on NO_2_. One possibility is that these activities are acting as a proxy for ventilation when occupants open windows and doors to release dust or smoke. However, these variables were based on self-reported activities after sampling and may be subject to recall bias.

The smart filter intervention suggested modest reductions in indoor PM_2.5_ concentrations. Nevertheless, results indicate that smart filters improved indoor air quality on average and have the potential to achieve greater reductions during periods of elevated outdoor PM_2.5_, including wildfire events, as has been demonstrated for other air filtration interventions [60]. Because no wildfire episodes occurred during the study period, smart filter performance under those high-exposure conditions could not be evaluated. Importantly, median indoor PM_2.5_ concentrations measured in study homes (∼14 *µ*g m^−3^ from UPAS measurements; see table 2) exceeded the current U.S. EPA annual guideline of 9 *µ*g m^−3^, highlighting the need to identify effective filtration and other strategies to reduce indoor PM_2.5_ exposures.

Overall, our findings suggest strong evidence that electric cooking can improve indoor air quality, while also noting that other studies and first principles still provide compelling evidence for the importance of sufficient ventilation, extractive kitchen hood use, and air filtration [8, 16, 17, 60, 61].

### 4.3. Exposure and health implications in study communities

The lower pollutant levels observed in CPUC program homes using electric stoves suggest meaningful air quality and corresponding health risk improvements for residents, who are rural, mostly low-income, and experience high baseline prevalence of asthma and other cardiopulmonary conditions [22, 25].

We present our results using U.S. EPA AQI [35] categories, which may be easier for impacted communities and non-experts to interpret, in figure 3 and table 3. The AQI provides a familiar way for the public to interpret pollutant levels against recognized thresholds. The amount of time indoor NO_2_ concentrations fell within different AQI categories during our measurement campaign revealed large contrasts between homes with different stove technologies. Gas stove homes had NO_2_ concentrations in the ‘Moderate’ category for 77 min d^-1^, on average, and in the ‘Unhealthy for Sensitive Groups’ category for nearly one hour per day. Indoor air quality exceeded the ‘Good’ range at least once in two out of three homes with gas stoves and reached the ‘Unhealthy for Sensitive Groups’ range in over 40% of homes with gas stoves. By comparison, NO_2_ in homes with electric stoves only reached the ‘Moderate’ range for 2 min d^-1^, on average, and never reached ‘Unhealthy for Sensitive Groups’. The nearly complete elimination of any NO_2_ AQI exceedances in electric stove homes strongly suggests that electrification interventions can meaningfully reduce respiratory health risks.

**Table 3.** Average daily minutes that indoor NO_2_ concentrations fell within each U.S. EPA AQI category, calculated using 1-h rolling averages of minute-level data. Values represent the mean across all homes by stove type, along with the percentage of homes experiencing at least one exceedance of each category during the intensive 48-h monitoring period.

AQI category [35]	Mean (min d^–1^)		Percent with ⩾1 exceedance	
	Electric homes (*n* = 66)	Gas homes (*n* = 52)	Electric homes (*n* = 66)	Gas homes (*n* = 52)
Good (0–53 ppb)	∼1314 min	∼1242 min	NA	NA
Moderate (54–100 ppb)	∼2 min	∼77 min	1%	67%
Unhealthy for Sensitive Groups (101–360 ppb)	0 min	∼52 min	0%	41%
Unhealthy (361–649 ppb)	0 min	∼2 min	0%	6%
Very Unhealthy (650–1249 ppb)	0 min	0 min	0%	0%

### 4.4. Monitoring approaches

Using a similar approach as presented by Tanner *et al* [62], we analyzed how increasing monitoring durations correlated with longer-term averages. Our evaluation demonstrates that relatively short campaigns can provide reliable estimates of longer-term NO_2_ and PM_2.5_ concentrations for indoor air. For NO_2_, cumulative daily time-averaged concentrations, when compared to 21-d average concentrations, had Pearson correlation >0.9 and bias <10% after just a few days. For PM_2.5_, a week of data was sufficient to approximate three-week averages with high stability. These findings suggest that while longer-term deployments provide more robust estimates of indoor pollutant concentrations, shorter deployments of a week or less can offer cost-effective and representative estimates, which can help scale studies across larger samples or multiple communities. These results also have practical implications for policy and program evaluation, where resources for monitoring are often constrained, and highlight the potential for targeted short-term sampling strategies to generate credible evidence.

Our application of a low-cost electrochemical NO_2_ sensor in the PAM also has implications for future monitoring efforts. At the group level, PAM-based NO_2_ estimates aligned well with the 405 reference monitor, and correlation was reasonably strong between the instruments (Pearson *r*-squared of 0.75, see scatterplot in figure S1); however, resolution and accuracy constraints of low-cost devices for estimating NO_2_ averages aligned with 24-h or annual standards can be problematic [63]. Still, their ability to capture combustion-related peaks (e.g., above 100 ppb), which were common in the gas homes (see figure 3), presents an opportunity. Asthma risks, for example, have been demonstrated to be strongly linked to shorter-duration exposure events [64–66], and low-cost sensors could play an important role in identifying health-relevant exposures for health-impacted households. There is an ongoing need to characterize the performance of low-cost NO_2_ and NO*_x_* sensors in the context of indoor air pollution measurement and to use the knowledge gained to refine monitoring approaches in a manner that enables efficient, scalable indoor air quality assessments in varied contexts.

### 4.5. Limitations

This study also has several limitations that should be noted. First, the cross-sectional design limits the ability to infer causal relationships between stove type and indoor pollutant concentrations; observed differences could reflect unmeasured household behaviors or characteristics not captured in our survey. Second, while we measured a set of key pollutants (NO_2_, NO, NO*_x_*, CO, CO_2,_ PM_2.5_, and BC), the scope was necessarily limited, and other potentially relevant pollutants, such as volatile organic compounds, were not included. Third, the study period did not coincide with significant wildfire smoke events, which prevented our ability to assess the smart filtration devices under the conditions they were designed to address. Finally, the study population (rural, low-income households in the San Joaquin Valley) represents an important, but specific context, and results may not be generalizable to other populations or housing types (e.g., urban, multifamily apartment buildings).

### 4.6. Policy implications and recommendations

The results from this study have direct implications for building, energy, and air quality policies. In California, for example, the Equitable Building Decarbonization Program (EBD) created under Assembly Bill 209 provides direct installs of cooking, heating, and cooling appliances at minimal or no cost to help low-income households in under-resourced communities. EBD will also provide incentives to accelerate the deployment of low-carbon building technologies across the state [67]. Our results suggest that this program should prioritize gas-to-electric stove replacements as a decarbonization measure that is likely to benefit indoor air quality.

The results also highlight the need to explicitly consider indoor air quality in regulatory frameworks: while outdoor NO_2_ levels are regulated, indoor exposures remain unaddressed, despite evidence that concentrations in homes with gas stoves frequently exceed health-based thresholds. The same is true for other criteria pollutants. Establishing and incorporating indoor air quality guidelines into housing codes, asthma prevention initiatives, and air quality management plans would help bridge this gap.

Equity considerations are central. Low-income households face financial and logistical (e.g., not having decision-making power when renting) barriers to appliance replacement, and rural households are often excluded from broader electrification initiatives. Programs like the CPUC San Joaquin Valley pilots and EBD’s Statewide Direct Install are well-aligned to address these equity issues. Other states and municipalities pursuing building decarbonization may also draw on our findings to support electrification policies that account explicitly for inequities in exposure to indoor air pollution and associated health burdens.

However, tenant protections are critical when programs incentivize or mandate electrification because, without safeguards, the benefits of cleaner appliances can be undermined by housing market dynamics. Landlords who receive subsidies or upgrade units may pass the costs onto tenants through rent increases or displacements [68]. Concrete protections could include (1) prohibiting rent increases tied directly to electrification upgrades and (2) barring tenant evictions for retrofit-related reasons. Incorporating tenant protections ensures that the intended public health and environmental benefits of electrification programs are equitably realized by vulnerable households rather than being offset by displacement or increased housing insecurity.

## Data Availability

The data cannot be made publicly available upon publication because the cost of preparing, depositing and hosting the data would be prohibitive within the terms of this research project. The data that support the findings of this study are available upon reasonable request from the authors. Supporting Methods and Data available at https://doi.org/10.1088/2752-5309/ae4ac9/data1.
